# TB-PRACTECAL: study protocol for a randomised, controlled, open-label, phase II–III trial to evaluate the safety and efficacy of regimens containing bedaquiline and pretomanid for the treatment of adult patients with pulmonary multidrug-resistant tuberculosis

**DOI:** 10.1186/s13063-022-06331-8

**Published:** 2022-06-13

**Authors:** Catherine Berry, Philipp du Cros, Katherine Fielding, Suzanne Gajewski, Emil Kazounis, Timothy D. McHugh, Corinne Merle, Ilaria Motta, David A. J. Moore, Bern-Thomas Nyang’wa

**Affiliations:** 1 Médecins sans Frontières, 10 Furnival Street, London, EC4A1AB UK; 2 Burnet Institute, 85 Commercial Rd, Melbourne, VIC 3004 Australia; 3 London School of Hygiene and Tropical Medicine, Keppel St, London, WC1E 7HT UK; 4 Swiss Tropical and Public Health Institute (affiliated with University of Basel), Kreuzstrasse 2, 4123 Allschwil, Switzerland; 5 UCL Centre for Clinical Microbiology, Royal Free Campus, UCL, Rowland Hill Street, NW3 2PF, London, UK; 6 The Special Programme for Research & Training in Tropical Diseases (TDR) World Health Organization, Avenue Appia 20, 1211, 27 Geneva, Switzerland; 7 TB Centre, London School of Hygiene and Tropical Medicine, Keppel St, London, WC1E 7HT UK; 8 Médecins sans Frontières, Plantage Middenlaan 14, 1018 DD Amsterdam, The Netherlands

**Keywords:** Multidrug-resistant tuberculosis, Bedaquiline, Linezolid, Clofazimine, Pretomanid, Moxifloxacin, Clinical trial, Phase 2/3, Multiarm multistage, RCT

## Abstract

**Background:**

Globally rifampicin-resistant tuberculosis disease affects around 460,000 people each year. Currently recommended regimens are 9–24 months duration, have poor efficacy and carry significant toxicity. A shorter, less toxic and more efficacious regimen would improve outcomes for people with rifampicin-resistant tuberculosis.

**Methods:**

TB-PRACTECAL is an open-label, randomised, controlled, phase II/III non-inferiority trial evaluating the safety and efficacy of 24-week regimens containing bedaquiline and pretomanid to treat rifampicin-resistant tuberculosis. Conducted in Uzbekistan, South Africa and Belarus, patients aged 15 and above with rifampicin-resistant pulmonary tuberculosis and requiring a new course of therapy were eligible for inclusion irrespective of HIV status. In the first stage, equivalent to a phase IIB trial, patients were randomly assigned one of four regimens, stratified by site. Investigational regimens include oral bedaquiline, pretomanid and linezolid. Additionally, two of the regimens also included moxifloxacin (arm 1) and clofazimine (arm 2) respectively. Treatment was administered under direct observation for 24 weeks in investigational arms and 36 to 96 weeks in the standard of care arm. The second stage of the study was equivalent to a phase III trial, investigating the safety and efficacy of the most promising regimen/s. The primary outcome was the percentage of unfavourable outcomes at 72 weeks post-randomisation. This was a composite of early treatment discontinuation, treatment failure, recurrence, lost-to-follow-up and death. The study is being conducted in accordance with ICH-GCP and full ethical approval was obtained from Médecins sans Frontières ethical review board, London School of Hygiene and Tropical Medicine ethical review board as well as ERBs and regulatory authorities at each site.

**Discussion:**

TB-PRACTECAL is an ambitious trial using adaptive design to accelerate regimen assessment and bring novel treatments that are effective and safe to patients quicker. The trial took a patient-centred approach, adapting to best practice guidelines throughout recruitment. The implementation faced significant challenges from the COVID-19 pandemic. The trial was terminated early for efficacy on the advice of the DSMB and will report on data collected up to the end of recruitment and, additionally, the planned final analysis at 72 weeks after the end of recruitment.

**Trial registration:**

Clinicaltrials.gov NCT02589782. Registered on 28 October 2015.

**Supplementary Information:**

The online version contains supplementary material available at 10.1186/s13063-022-06331-8.

## Administrative information

Note: the numbers in curly brackets in this protocol refer to SPIRIT checklist item numbers. The order of the items has been modified to group similar items (see http://www.equator-network.org/reporting-guidelines/spirit-2013-statement-defining-standard-protocol-items-for-clinical-trials/).

**Table:** 

Title {1}	A randomised, controlled, open-label, phase II–III trial to evaluate the safety and efficacy of regimens containing bedaquiline and pretomanid for the treatment of adult patients with pulmonary multidrug-resistant tuberculosis (TB-PRACTECAL)
Trial registration {2a and 2b}.	Clinicaltrials.gov registration number NCT02589782
Protocol version {3}	Version 7.0 of 13 July 2020 (South Africa, inclusion criteria ≥ 15 years) Version 7.1 of 14 August 2020 (Belarus and Uzbekistan, inclusion criteria ≥18 years)
Funding {4}	Médecins sans Frontières
Author details {5a}	• Médecins Sans Frontières • London School of Hygiene and Tropical Medicine, London, UK • University College London, London, UK • Swiss Tropical and Public Health Institute • Burnet Institute, Melbourne, Australia • The Special Programme for Research and Training in Tropical Diseases (TDR), World Health Organization.
Name and contact information for the trial sponsor {5b}	Bern-Thomas Nyang’wa, Plantage Middenlaan 14, 1018 DD, Amsterdam, The Netherlands Bern.nyangwa@london.msf.org +44(0) 788 580 4202
Role of sponsor {5c}	Médecins sans Frontières as sponsor, is responsible for the design, collection, trial management and has final authority over submission. Data analysis will be performed by LSHTM.

## Introduction

### Background and rationale {6a}

The emergence of rifampicin-resistant tuberculosis (RR-TB), defined as TB caused by strains of *Mycobacterium tuberculosis* (MTB) resistant to at least rifampicin (R), has complicated global efforts to control the TB epidemic. Approximately half a million cases of RR-TB occur in the world annually, representing about 6% of the world’s annual TB burden. Currently, around 38% of people with RR-TB are initiated on treatment and there is an urgent need to scale up treatment programmes [1]. Scale-up is being severely hampered by financial, political, logistical, and technical obstacles and one of the most important challenges is the current standard of care (SOC) [2]. The study was initially conceived as targeting multidrug-resistant tuberculosis (MDR-TB) which indicates additional resistance to isoniazid (H); however, as current treatments for MDR-TB and RR-TB are the same, the two terms in this protocol can be considered interchangeably. Additionally, since this study was conceived, the definition of extensively drug-resistant TB has been updated and is now known as pre-XDR [3]. The protocol refers to the pre-2021 definition of XDR.

The current treatment regimen used to treat RR-TB has poor efficacy. In a recent individual patient meta-analysis of treatment outcomes for pulmonary RR TB, only 61% of patients had successful outcomes, whilst 16% were lost to follow up and 14% died [4]. This poor effectiveness combined with high costs and implementation challenges, prevents many national TB programmes from offering treatment for MDR-TB [5]. This in turn fuels the spread of RR-TB infections [6]. There is clearly a global need for an improved treatment regimen for RR-TB that is efficacious, safe, tolerable, and that can be implemented quickly in a variety of geographic, epidemiologic, and programmatic settings. Given the high rates of HIV co-infection among certain populations of patients with RR-TB [7], it is imperative that patients with HIV be included in any new treatment regimen strategies.

Recently, several new anti-tuberculosis agents have been developed or re-purposed, including bedaquiline (TMC207; B), delamanid (OPC-67683, D), pretomanid (PA-824; Pa) and the oxazolidinones, including linezolid (Lzd). These agents each act upon a completely novel target in the tuberculosis bacillus and have the potential to prove highly effective, especially when combined with one another and with existing antituberculosis drugs. In addition, there is promising evidence from phase II clinical trials for some of these new drugs when used with existing anti-tuberculosis drugs [8, 9]. Shortened treatment regimens have been explored in phase III trials using existing antituberculosis medications in novel combinations (STREAM study) [10] and in several ongoing trials [11].

TB-PRACTECAL is evaluating novel combinations of new and existing antituberculosis drugs in a 6-month, all-oral treatment regimen for safety and efficacy outcomes. Regimens have been selected for their potential efficacy, safety and ease of implementation in the field.

### Objectives {7}

#### Primary objectives

Stage 1

Identify regimens containing bedaquiline and pretomanid for further evaluation based on safety and efficacy outcomes after 8 weeks of treatment.

Stage 2

Evaluate the safety and efficacy of the investigational regimens containing bedaquiline and pretomanid compared with the SOC at 72 weeks post-randomisation.

#### Secondary objectives

Stage 1

1. To compare the frequency of serious adverse events (SAE), and grade 3 and higher adverse events (AE’s).

Stage 2

1. To compare the rates of culture conversion in liquid media between the SOC and investigational arms at specified time periods after randomisation (i.e. 12 weeks, 24 weeks);
2. To compare the frequency of SAEs, grade 3 and higher AEs between the SOC arm and investigational arms; and
3. To compare unfavourable outcomes between the SOC arm and investigational arms (including failure, treatment discontinuation, death, loss to follow-up, still on treatment at 108 weeks and recurrence) at specified time periods post randomisation (i.e. 24 weeks, 48 weeks and 108 weeks).

## Trial design {8}

This is a multi-centre, open-label, multi-arm, randomised, controlled, phase II-III trial; evaluating short treatment regimens containing bedaquiline and pretomanid in combination with existing and re-purposed anti-TB drugs for the treatment of biologically confirmed pulmonary multidrug-resistant TB (MDR-TB).

The study is divided into two stages, with a seamless transition between the stages, meaning recruitment into an arm will only stop after a decision has been taken following stage 1 primary endpoint data analysis. Each randomised patient will complete his/her allocated treatment unless an unfavourable endpoint is reached. All recruited patients will be followed up for 108 weeks post randomisation unless they die, withdraw consent earlier or are censored at no earlier than 72 weeks. The locally approved SOC regimen for MDR-TB is used as the internal control for both safety and efficacy.

The first stage corresponds to a Phase II trial of safety and preliminary efficacy in patients with MDR-TB. Patients are recruited into 3 parallel bedaquiline (B) and pretomanid (Pa) containing regimen arms plus a SOC control. The main objective of stage 1 is to select drug regimens for evaluation in stage 2 based on 8-week safety and efficacy endpoints. All stage 1 patients undergo intensive cardiological evaluations to establish the early QT-specific liability of the regimens, and also closely monitor for early haematological and hepatic events.

Investigational arms that do not meet predefined safety and efficacy criteria (percent of culture conversion > 40%; percent of unfavourable outcomes <45%) are not considered for further evaluation. The arms that meet these pre-defined safety and/or efficacy criteria will be eligible to be evaluated for long-term safety, tolerability and efficacy in stage 2.

If fewer than two arms are available for stage two assessments, the Scientific Advisory Committee (SAC) makes recommendations on whether new arms should be introduced in the study. If more than two investigational arms are available for the stage 2 assessment, the SAC makes recommendations on which two arms are eligible to be taken forward to the trial steering committee.

The second stage corresponds to a phase III trial. Patients in this stage were to be recruited into up to 2 arms chosen from stage 1 plus the SOC. The regimens are primarily evaluated for efficacy in comparison with the SOC arm at 72 weeks post-randomisation. The primary efficacy outcome in stage 2 is a composite endpoint of the percentage of unfavourable outcomes (see section 7.1 for outcome definitions). Secondary outcomes include safety outcomes, and in particular, the percentage of patients experiencing SAEs and/or Grade 3 or 4 AEs during the treatment.

Stage 1 patients enrolled in arms that are continued to stage 2 are included in the sample size for stage 2. After the last enrolled patient has reached 72 weeks, all patients who have not reached their secondary endpoint are to be censored.

## Methods: Participants, interventions and outcomes

### Study setting {9}

The study is conducted in seven trial sites, in three countries. In Uzbekistan, the trial is implemented in four rayons of Karakalpakstan and Tashkent city. In Karakalpakstan, each of these rayons has a central clinic and several directly observed therapy (DOT) corners where trial patients get ambulatory care. Hospitalisation of trial participants (for severely ill patients or per local procedures) is in the Republican Specialised Scientific-Practical Medical Centre for Phyisiology and Pulmonology hospital in Tashkent City or Nukus TB2 hospital in Karakalpakstan. In Kwa-Zulu Natal province of South Africa, patients are hospitalised in Doris Goodwin, Don McKenzie and King Dinuzulu Hospitals, and in Gauteng province, the trial is conducted in Helen Joseph Hospital. In Belarus, the trial is conducted in Minsk City and Oblast. Participants are primarily followed up and hospitalised at the Republican Scientific and Practical Centre for Pulmonology and Tuberculosis hospital.

### Eligibility criteria {10}

#### Inclusion criteria

Patients eligible for inclusion in the trial fulfiled all of the following criteria:

- Male or female patients aged 15 years or above (where locally approved), regardless of HIV status;
- Microbiological test (molecular or phenotypic) confirming the presence of *M. tuberculosis* in sputum;
- Resistant to at least rifampicin by either molecular or phenotypic drug susceptibility test; and
- Completed informed consent form (ICF).

#### Exclusion criteria

Patients were not eligible for inclusion in the trial if they meet any of the following criteria:

- Known allergies, hypersensitivity, or intolerance to any of the study drugs;
- Pregnant, breast-feeding, or unwilling to use appropriate contraceptive measures if of childbearing potential;
- Alanine transaminase (ALT) and/or aspartate transaminase (AST) and/or bilirubin >3 times the upper limit of normal;
- Taking any medications contraindicated with the medicines in the trial;
- Fredericia corrected QT interval (QTcF) > 450 ms;
- One or more risk factors for QTc prolongation (excluding age and gender) or other uncorrected risk factors for torsades de pointes (TdP);
- History of cardiac disease, syncopal episodes, symptomatic or significant asymptomatic arrhythmias (with the exception of sinus arrhythmia);
- Any baseline laboratory value consistent with Grade 4 toxicity;
- Moribund;
- Known resistance to bedaquiline, pretomanid, linezolid or delamanid;
- Any other condition (social or medical) which, in the opinion of the investigator, would make study participation unsafe;
- Prior use of bedaquiline and/or pretomanid and/or linezolid and/or delamanid for one or more months;
- Patients not eligible to start a new course of MDR-TB/ XDR TB treatment according to local protocol, including but not limited to:
  - currently on MDR-TB treatment for at least 2 weeks (and not failing),
  - no permanent physical address,
  - loss to follow-up in previous treatment with no change in circumstance and motivation.
- Tuberculous meningoencephalitis, brain abscess, osteomyelitis or arthritis.

### Who will take informed consent? {26a}

An ICF in clear, simple language is provided to the patient. The investigator collects written consent from each patient before any study-specific procedure is conducted. Two original ICFs are completed, dated and signed personally by the patient and by the investigator. The patient is given one signed original form; the second original is kept by the investigator.

If the patient is unable to read, a relative or an impartial witness is present during the informed consent discussion. The patient gives consent orally and, if capable of doing so, completes, signs (or thumbprints) and personally dates the information and consent form. The witness then completes, signs and dates the form together with the investigator.

For individuals under the legal adult age, both the patient and legal guardian must fully understand and agree to participate. An assent is signed by the patient as well as an ICF by the legal guardian prior to screening.

All ICF documents and supporting patient materials are approved by the local ethics committee.

### Additional consent provisions for collection and use of participant data and biological specimens {26b}

Separate consent procedures and forms are used for participation in the trial sub-studies [12–14].

## Interventions

### Explanation for the choice of comparators {6b}

The comparator is the locally approved SOC which is as much as possible consistent with the WHO recommendations for the treatment of RR-TB. The regimen chosen varies depending on the country as well as over time to ensure those randomised to this regimen could access the best available care. For longer regimens, treatment is individualised with the constituent drugs changing depending on the proven or expected drug susceptibility testing (DST) of the infecting bacilli. The algorithm is described in the country-specific clinical guidelines, implemented alongside protocol v 7.0/7.1 and includes the use of at least four drugs including bedaquiline (B), a later-generation quinolone - moxifloxacin (Mfx) or levofloxacin (Lfx), linezolid (Lzd), clofazimine (Cfz), pyrazinamide (Z), prothionamide/ ethionamide (Pto/Eto) or cycloserine (Cs)/ terizidone (Trd). Other drugs such as amikacin, ethambutol (E), high-dose isoniazid, delamanid, para-aminosalicylic acid (PAS), imipenem/cilastatin and meropenem may also be used. A standardised shorter regimen or modified shorter regimen for RR-TB patients with no second-line drug resistance may be used if approved locally (Table 1).

**Table 1:** Standard of care drugs and dosing

Drug	Recommended dose by weight							
	30–35 kg	36–40 kg	41–45 kg	46–50 kg	51–55 kg	56–60 kg	61–70 kg	>70 kg
Isoniazid (high dose)	By weight, 15 mg/kg. Max 600 mg							
Ethambutol	800mg	800mg	800mg	800mg	1200mg	1200mg	1200mg	1200mg
Pyrazinamide (20–30 mg/kg) Max 2000 mg	800 mg	800 mg	1200 mg	1200 mg	1600 mg	1600 mg	1600 mg	2000 mg
Amikacin	500 mg	500 mg	750 mg	750 mg	1000 mg	1000 mg	1000 mg	1000 mg
Levofloxacin	750 mg	750 mg	750 mg	750 mg	1000 mg	1000 mg	1000 mg	1000 mg
Moxifloxacin	400 mg	400 mg	400 mg	400 mg	400 mg	400 mg	400 mg	400 mg
Ethionamide/prothionamide	500 mg	500 mg	500 mg	500 mg	750 mg	750 mg	750 mg	750 mg
Terizidone/cycloserine	By weight (15–20 mg/kg)	750 mg	750 mg	750 mg	750 mg	750 mg	750 mg	750 mg
Para-aminosalicylic acid	4 g	8 g	8 g	8 g	8 g	8 g	8 g	8 g
Clofazimine	100 mg							
Linezolid	300 mg	600 mg	600 mg	600 mg	600 mg	600 mg	600 mg	600 mg
Bedaquiline	400mg once daily for 2 weeks then 200mg three times a week							
Delamanid	100 mg twice daily							
Imipenem/cilastatin	1000 mg imipenem/1000 mg cilastatin every 12 h							
Amoxicillin/clavulanate	500/125mg twice daily (*ONLY* for use in combination with Imipenem / cilastatin, give orally 30min before infusion)							

### Intervention description {11a}

Investigational regimens in stage 1:

- Regimen 1: bedaquiline + pretomanid + linezolid + moxifloxacin for 24 weeks
- Regimen 2: bedaquiline + pretomanid + linezolid + clofazimine for 24 weeks
- Regimen 3: bedaquiline + pretomanid + linezolid for 24 weeks

Investigational regimen in stage 2 (Table 2):

- Regimen 1: bedaquiline (B) + pretomanid (Pa) + linezolid (Lzd) + moxifloxacin (Mfx) for 24 weeks

**Table 2:** Investigational regimen drugs and dosing

Bedaquiline	400 mg once daily for 2 weeks followed by 200 mg 3 times per week for 22 weeks
Pretomanid	200mg once daily for 24 weeks
Moxifloxacin	400 mg once daily for 24 weeks
Linezolid	600mg daily for 16 weeks then 300mg daily for the remaining 8 weeks (or earlier when moderately tolerated)
Clofazimine	50 mg (less than 33 kg), 100 mg (more than 33 kg) for 24 weeks

### Criteria for discontinuing or modifying allocated interventions {11b}

#### Treatment interruptions

Patients may interrupt/pause treatment for up to 14 consecutive days and be able to restart. This may result from the investigator temporarily withholding the treatment due to an adverse event or other social/logistical reasons. After sufficient recovery and strictly in line with the current version of the TB-PRACTECAL Clinical Guidelines, the patient may be restarted on the same treatment following consultation with the medical monitor.

Patients may also miss treatment due to challenges with adherence. The investigator and trial team support the patient in identifying any underlying causes. Up to 14 consecutive days can be missed and treatment recommenced. If the patient misses greater than 14 consecutive days or is adjudged to have poor adherence as defined in TB-PRACTECAL Clinical Guidelines, they should permanently discontinue treatment. If treatment discontinuation is the final outcome, the investigator, with the support of the medical monitor, is responsible for linking the patient to further care.

Patients missing some days during the treatment phase should extend the treatment phase by the number of days missed. In this case, the last visit of the treatment period should be delayed to the date of the last dose.

#### Discontinuation and withdrawal criteria

Patients must discontinue study treatment, whatever trial regimen they have been allocated to, with any of the following events:

- Grade 3 or higher QT prolongation and other cardiac rhythm disturbances
- Grade 3 or higher hearing loss
- Patients who are felt to be non-adherent by the Investigator as evidenced by missing more than 2 consecutive weeks of treatment or meeting criteria outlined in the Clinical Guidelines.
- Patients who withdraw consent
- Permanently stopping or adding at least one drug in an investigational arm or two drugs in the SOC. Dose reduction or short holidays of less than 2 weeks will not be considered as significant modifications. Restarting treatment should only be done with the explicit recommendation from the Medical Monitor.
- At the discretion of the Investigator, a patient may discontinue treatment in case of any adverse event, laboratory abnormality, or intercurrent illness which, in the judgement of the Investigator, presents a substantial clinical risk to the subject with continued study regimens use.

If a patient's study regimen must be discontinued before the end of the treatment regimen, this will not result in automatic withdrawal of the patient from the study. Patients who discontinue treatment will be followed up to week 108, guided by the investigational schedule, unless they withdraw consent.

The management of patients who become pregnant whilst taking study drugs varies by site. In Belarus and Uzbekistan, patients who become pregnant and wish to continue their pregnancy are discontinued from the trial and are offered a regimen in line with national guidelines. In South Africa, patients and investigators are able to make individualized decisions in conjunction with the medical monitor whether to continue on the study regimen. All pregnancies are reportable to pharmacovigilance.

### Strategies to improve adherence to interventions {11c}

All study treatments are delivered either through directly observed therapy (DOT) or video observed therapy (VOT). Treatment is delivered under direct observation by treatment supporters or nurses in health facilities, in patient homes or other community settings convenient to patients. Treatment is administered and observed daily 7 days a week in the investigational arms and at least 6 days a week in the SOC. The responsible study nurse or treatment supporter will be in charge of receiving the study drugs from the trial pharmacist, checking that patients receive the correct regimen and documentation of observed drug intake. Data on adherence and pill intake will be recorded on standardised forms and in the electronic case report form (eCRF).

Counselling and social support tailored to site needs as well as timely identification and management of adverse events are also key adherence support activities mandated by the sponsor.

### Relevant concomitant care permitted or prohibited during the trial {11d}

All therapies (prescriptions or over-the-counter medications, including vitamins and herbal supplements) different from the trial drugs are recorded in the concomitant therapy section of the eCRF.

#### Prohibited drugs/absolute contraindications

The following therapies are not allowed during the trial: efavirenz; drugs known to significantly prolong the QTc interval, including neuroleptics-phenothiazines, quinoline antimalarials, anti-arrhythmic drugs and fluoroquinolones other than those included in the trial regimens; drugs that may induce muscle damage such as HMG-CoA reductase inhibitors; strong CYP3A4 inducers; strong CYP3A4 inhibitors for more than 2 weeks; mono-amine oxidase inhibitors; drugs known to induce myelosuppression. Should any of the above-listed medication be administered concomitantly to study drugs, this is considered a protocol deviation.

#### Relative contraindicated medications

The following drugs have either established or suspected interactions or overlapping toxicities with the trial drugs. Therefore, their use should only be considered in situations where alternative options are either not available or are riskier than the administration of these drugs. Closer follow-up of patients taking these drugs is recommended. Site principal investigators should consider consulting the Sponsor Medical Monitor before prescribing them. Relatively contraindicated medications include antiretroviral medications, such as protease inhibitors, zidovudine and abacavir, selective serotonin reuptake inhibitors, tricyclic antidepressants and drugs known to cause limited QTc prolongation e.g. metoclopramide.

### Provisions for post-trial care {30}

Patients who discontinue study treatment for any reason except if lost to follow-up will be offered an alternative, individualized rescue treatment based on their clinical condition and the latest drug susceptibility testing results and in line with national recommendations of the country. The rescue regimen is at the discretion of the Investigator in accordance with local standards and may include registered drugs accessible only through the trial. Investigators are encouraged to discuss the management of these patients with the Medical Monitor. Patients may also elect to have rescue treatment through their local tuberculosis programme.

Patients who discontinue treatment are encouraged to complete visits as much as possible per the investigational schedule (including SOC) unless consent is withdrawn. Continue all safety investigations as much as possible per investigational schedule up to week 108 post-randomisation and document all findings in the patient’s file.

Following the discontinuation visit, sputum submissions, HIV tests, viral load and CD4 counts are no longer required for trial purposes. However, if performed for ongoing clinical management then the results should be requested and documented in the patient’s file. TB drugs prescribed to the patient as part of a rescue treatment regimen are not considered investigational medical product.

### Outcomes {12}

#### Stage 1 primary outcomes

- Efficacy outcome: percentage of patients with culture conversion in liquid media at 8 weeks post-randomisation.
- Safety Outcome: percentage of patients with treatment discontinuation and death at 8 weeks post-randomisation.

#### Stage 1 secondary outcomes

- Percentage of patients with grade 3 or higher QTc prolongation within 8 weeks post-randomisation
- Percentage of patients experiencing at least one SAE within 8 weeks post-randomisation
- Percentage of patients experiencing at least one new Grade 3 or higher AE within 8 weeks post-randomisation

#### Stage 2 primary outcome

- Percentage of patients with an unfavourable outcome at 72 weeks post-randomisation.

#### Stage 2 Secondary outcomes

- Percentage of patients with culture conversion at 12 weeks post-randomisation
- Median time to culture conversion
- Percentage of patients with an unfavourable outcome at 24 weeks post-randomisation
- Percentage of patients with an unfavourable outcome at 108 weeks post-randomisation
- Percentage of patients with SAEs or new Grade 3 or higher AEs at the end of treatment (at 24 weeks in investigational arms and at 80+ weeks in SOC arm)
- Percentage of patients with SAEs or new Grade 3 or higher AEs at 72 weeks post-randomisation
- Percentage of patients with SAEs or new Grade 3 or higher AEs at 108 weeks post-randomisation
- Mean single change in QTcF at 24 weeks post-randomisation
- Percentage of patients experiencing recurrence by week 48 in investigational arms (Table 3)

**Table 3:** Study outcome definitions

**Death:** Death of a patient from all causes.	
**Treatment failure in standard of care arm:** *Conventional MDR-TB regimen* The presence of a positive mycobacterial culture in MGIT liquid media in each of two separate specimens taken at least four weeks apart (+/− 2 weeks) from week 28 until week 108 *Shorter MDR-TB regimen* The presence of a positive mycobacterial culture in MGIT liquid media in each of two separate specimens taken at least four weeks apart from week 16 (+/− 2 weeks) or later	
**Treatment failure in investigational arms:** The presence of a positive culture in MGIT liquid media in each of two separate specimens taken at least four weeks apart from week 16 (+/− 2 weeks) or later.	
**Lost-to-Follow-up:** A patient who has missed his/her appointment after completing treatment and cannot be traced until the end of the expected follow-up period (108 weeks or at time of censure).	
**Treatment discontinuation:** A decision by an investigator to discontinue treatment: 1) either due to the need to significantly modify the trial regimen for whatever reason, 2) or due to the patient missing some or all drugs regularly 3) or due to the patient missing all drugs for more than 2 consecutive weeks	
**Still on treatment:** A subject who is still taking treatment for M/XDR-TB 108 weeks after starting but hasn’t been declared as treatment failure.	
**Culture conversion:** At least two consecutive negative sputum cultures taken 4 weeks apart (+/− 2 weeks). The date of the first negative culture will be considered the conversion date.	
**Recurrence :** A subject who has completed treatment without being declared a failure and who has subsequently been diagnosed and require MDR-TB treatment (for whom there is evidence that the recurrence is due to an MDR or XDR TB strain)	
**Re-infection:** A subject who has completed treatment without being declared a failure and who has subsequently been diagnosed and require MDR-TB treatment but for whom there is evidence that the recurrence is due to a different strain to the baseline specimen. If the strain is a DS strain the patient is subsequently non-assessable.	
**Relapse:** A subject who has completed treatment without being declared a failure and who has subsequently been diagnosed and require MDR-TB treatment and for whom there is evidence that the recurrence is due to the same strain recorded in the baseline specimen.	
**Unfavourable outcome:** A composite outcome comprising death, treatment failure, treatment discontinuation, loss to follow up, still on treatment at 108 weeks and recurrence.	

### Participant timeline {13}

The trial visits are divided into screening, inclusion, week 1–8 (stage 1 and stage 2 differing investigations), week 9–24 (investigational and SOC arms similar investigations) and week 25–108 (investigational and SOC arms differing investigations). Different visit windows apply for the treatment and follow-up period as follows: +/− 1 day for visits in the first 2 weeks, +/− 3 days for weekly visits and +/− 7 days for 4 or 8 weekly visits. Day 0 is defined as the day of randomisation. The inclusion visit may be done on the same day or a day earlier. Study visits in the first two weeks will be based on the day treatment was actually started and subsequent weekly visits are defined as seven-day multiples from that point. Trial investigational schedule schematic for stage 1 is described in Additional file 1.

### Sample size {14}

The analysis of stage 1 is based on test arms only and there is no comparison with the SOC arm. Therefore, the sample size is based on the number required to detect culture conversion < 40% and/or a percentage of treatment discontinuation for any cause and death >45% in an investigational arm.

With 60 participants in an investigational arm evaluable for treatment discontinuation, 29% [15] patients or fewer would need to discontinue, to have 80% power with a one-sided alpha=0.05 to reject the null hypothesis of a true underlying discontinuation rate of 45% (or greater). (Sample size determination for one proportion {u(√[π(1- π)] +v√[π0(1- π0)]}2/( π- π0)2, *u*=1-power, *v*=two-sided significance level).

Similarly, if there are 29% or fewer discontinuations, there would be 43–60 patients remaining per arm to evaluate culture conversion. In this scenario, 55% (33/60)–58% (24/43) of the patients would need to have culture conversion to have 80% power with a relaxed one-sided alpha=0.075 to reject the null hypothesis of a true underlying conversion rate of 40% (or lower).

Analysis at stage 2 is based on a non-inferiority design to assess efficacy. Sample size calculations are based on this efficacy non-inferiority comparison of the composite primary outcome. In order to allow for both the adaptive nature of the design and the multiple comparisons with three possible arms, an alpha of 1.7% was used.

The underlying assumptions for these power calculations are based on the failure rates seen in patients receiving the control regimen at the time of original protocol writing. An analysis of loss to follow up (LTFU) over time suggested an additional 10% LTFU rate per 6 months of treatment after the first 6-8 months. These data were also supported by a large individual patient data meta-analysis of more than 9000 MDR-TB patients [3]. If assumed that the control and investigational regimens perform the same on all variables included in the composite efficacy other than LTFU, then the likely decrease in LTFU rate expected in the investigational arms due to the shorter length of treatment would lead to the investigational arm performing better overall. Although the primary outcome is efficacy at 72 weeks, the final sample size allows for adequate power to assess the secondary outcome of efficacy at 108 weeks.

Therefore, assuming a failure rate of 50% in the control arm and of 45% in the investigational arms, 181 patients per arm would be needed for a delta of 12% with approximately 85% power and a one-sided 98.3% confidence interval (to allow for both the adaptive nature of the design and the multiple comparisons of the three arms).

The delta of 12% was chosen following extensive consultation. The benefits of reducing treatment duration from 9-24 months to 6 months, reduced pill burden, and all oral nature of the investigational regimens have considerable advantages which would outweigh a possible increase in failure rate as reflected in the 12% non-inferiority margin. This delta is also comparable to contemporary ongoing MDR-TB clinical trials which have been approved by the US Food and Drug Administration (FDA) and local regulatory agencies [9].

Information available from patients recruited by the end of stage 1 suggested that the number excluded from the modified intention to treat (mITT) population is closer to 10% and therefore the recruitment target was increased to 201 per arm.

### Recruitment {15}

Patients in the catchment areas with a molecular WHO-approved rapid diagnostic test (WRDT) showing rifampicin resistance were assessed for eligibility by investigators in liaison with local clinics. Patients with sputum cultures showing rifampicin resistance or who were not responding to their current treatment could also be referred. Patients fitting initial eligibility criteria were invited to counselling sessions and after full informed consent, could be included in the study.

A community engagement strategy was developed that described the overall objectives, implementation and monitoring of trial community engagement activities. From this, in consultation with local stakeholders, context-adapted community engagement plans were developed.

The aims of these plans were (i) to engage in a two-way dialogue to harness local knowledge and patient insights towards better trial preparation, recruitment and retention of participants and (ii) to build a positive foundation of understanding, acceptance, goodwill and support in order to identify and overcome barriers to participation. These plans laid the groundwork for the models of care to deliver patient-centred care and cement partnerships with local TB providers. These plans are continuously reviewed and updated in response to recruitment challenges.

Additionally, expansion of trial catchment areas and new trial sites were added when recruitment was slower than anticipated.

Assignment of interventions: allocation

### Sequence generation {16a}

Treatment allocation was done using ratios of 1:1:1:1 in stage 1 and 1:1 in stage 2. Randomisation lists were produced by the trial statistician for each stage of the study, stratified by study site. For stage 1 randomisation, the “ralloc” package in Stata [16] was used to create randomisation lists for each site (with block sizes of 8).

In stage 2, the sequence was generated by proprietary software also used to undertake the randomisation [17]. In stage 2, varying block sizes of 4 and 6 were used.

### Concealment mechanism {16b}

In stage 1, the code for each individual was provided in a secure manner to the sites in separate, opaque sealed envelopes and assigned to individuals in the order in which they were enrolled in the study. The sealed randomisation envelopes look identical and were kept in a separate room, in a locked cupboard with restricted access. Each envelope had a sequential number and contained the details of the regimen the patient would receive. The randomisation list was kept by the trial statistician.

### Implementation {16c}

The allocation sequence was generated by the trial statistician and envelopes (stage 1) or by the randomisation system (stage 2) provided to the sites. Randomisation was undertaken according to the local SOP at the request of an investigator, once all screening and inclusion activities were complete. Personnel in charge of the randomisation, as well as the witness, were not involved in direct patient care. In stage 1, delegated personnel were responsible for opening the next sequential envelope, documenting the treatment allocation and assigning the study number. In stage 2, the same procedure was followed except randomisation personnel used an online, self-service randomisation system to receive the treatment allocation in lieu of envelopes [17]. Randomisation personnel then notified the investigator of the allocation.

Assignment of interventions: blinding

### Who will be blinded {17a}

TB-PRATECAL is an open-label trial; however, the laboratory personnel and centralised electrocardiogram (ECG) reviewers are blinded to treatment allocation.

### Procedure for unblinding if needed {17b}

Not clinically applicable.

## Data collection and management

### Plans for assessment and collection of outcomes {18a}

All study data are first recorded in source documents before being transcribed in the eCRF [15]. Radiology, ophthalmology, and audiometry data are acquired and recorded by the sites in sponsor developed forms and interpreted locally. ECGs are transmitted by the sites to a central ECG laboratory to undergo quality checks and blinded central review and reporting. Laboratory data is recorded onto the quality forms contained in the mycobacteriology and safety quality manuals before being transcribed into the eCRF. Where a laboratory information system conforming to the Code of Federal Regulations Title 21, Part 11 (21 CFR Part 11) requirement is available, the data will be transmitted directly from the laboratory information system into the clinical database.

The designated source documents which are agreed between the sponsor and the investigators at each site are available at the trial site, to allow retrospective checks that source data have been accurately and completely transcribed into the eCRF.

### Plans to promote participant retention and complete follow-up {18b}

Retention in care is within the scope of the community engagement plan through activities to build mutual trust and respect between study staff and participants. Along with home-based care (in Uzbekistan), DOT and VOT tools, adherence guidelines have been designed according to the site needs. Individual and group counselling is available for participants throughout treatment and follow-up. An individualised strategy to meet patient needs has been put in place in all sites (transportation to the facility, follow up through secure social media, convenient appointments, engagement of social supports in adherence). In the event of missed visits or challenges with treatment adherence are identified, the study team makes every effort to trace the patient.

### Data management {19}

An eCRF was designed to record all the data collected as per the protocol. An eCRF is completed for each participant. The eCRF, together with all trial related forms and logs are produced by the sponsor. The eCRFs have been built using OpenClinica [15], a fully validated secure web-enabled software that conforms to 21 CFR Part 11 requirements.

The delegated investigator staff enter the data required by the protocol, but the Principal Investigator is responsible for assuring that the data entered into the eCRF are complete, accurate, and consistent with the source documents and that entry and updates are performed in a timely manner. Corrections and alterations of data on the eCRF or source documents must be made by the investigator or by the delegated person from his/her team, dated and signed. Changes to the eCRF are tracked electronically in the database audit trail.

Adverse events are coded using the Medical dictionary for regulatory activities (MedDRA) terminology [18]. Concomitant medications are coded using international non-proprietary names (INN) [19].

The Data Manager, or their delegate, reviews the eCRF data entered by investigator staff for completeness and accuracy. Edit checks are built into the eCRF and contain univariate checks on the eCRF including missing values in required fields, range checks and valid values among others. Electronic data queries stating the nature of the problem and requesting clarification are created for discrepancies and missing values and sent to the investigational site via the electronic data capture system. Details are documented in the TB-PRACTECAL Data Management Plan.

Once the trial data has been verified for completeness and accuracy, the database will be locked in compliance with the database locking standard operating procedure (SOP).

### Confidentiality {27}

The Principal Investigator (or delegate) is responsible for recording the patient’s personal details, screening number and unique trial number in the subjects’ identification list. This list is kept in a lockable safe in the trial office, with access restricted to authorised trial staff only. All laboratory specimens, including stored specimens, as well as trial reports, data collection tools, and administrative forms are only identified by using the patient’s unique trial number. Names are not used on any of these documents. All local databases are secured with password-protected access systems. The Investigator ensures anonymity of the patient and that all documents are anonymised before being transmitted to the sponsor.

### Plans for collection, laboratory evaluation and storage of biological specimens for genetic or molecular analysis in this trial/future use {33}

WRDT testing will be used to screen for eligibility. Those participants with TB isolates resistant to rifampicin by the rapid molecular tests will then be evaluated by MGIT drug sensitivity testing (DST) for confirmation of MDR-TB. Liquid culture will be done using the MGIT 960 system [20]. Rapid testing will be done according to site-specific SOPs as detailed in the Mycobacteriology Laboratory Manual.

Two sputum samples (1 early morning and 1 coached spot expectoration sample) will be collected from trial participants at least once in a month during investigational arms’ treatment and once every two months during follow-up. DSTs will be performed on pure cultures from specimens obtained at baseline, during treatment and follow-up period, using MGIT. Susceptibility to the following drugs will be tested at baseline and from week 16 onwards if culture positive: H, R, E, Z, S (streptomycin), Km (kanamycin), Cm (capreomycin), Mfx and/or Ofx (ofloxacin). Culture isolates at the same intervals as above will be stored for minimum inhibitory concentration (MIC) determination for B, Pa, Lzd, Cfz +/− Mfx when indicated.

Mycobacterial DNA will be stored at baseline (D0, D7 and at W4 if the D0 and D7 DNA samples are not available) from all patients. In patients who revert after culture conversion or develop recurrent TB during the follow-up period after the end of TB treatment, genotyping will be performed on paired *M. tuberculosis* positive isolates (originating from that patient), in order to differentiate relapse and reversion from re-infection. Isolate DNA for such testing will be stored at the site and shipped to approved testing centres according to site-specific SOPs. If exportation of biological material is not allowed, then genotyping may be performed on site.

Refer to the TB-PRACTECAL Mycobacteriology Laboratory Manual for details of the standard procedures for the key methodologies, quality control practices, interpretation of findings and standardised terminology. The laboratory team will be blinded to the trial arm of the participants at all times when processing the samples.

All specimens planned for further analysis in sub-studies are detailed in the sub-study protocol [14].

## Statistical methods

### Statistical methods for primary and secondary outcomes {20a}

Demographic and baseline characteristics of the randomised patients will be summarised by treatment arm. The distribution of categorical variables will be summarised by counts and percentages. Quantitative variables will be summarised using the mean and standard deviation (SD) or median and inter-quartile range (IQR), where appropriate, and the minimum and maximum and sample size of non-missing data. Any imbalances of baseline characteristics across treatment arms identified through examination of these summaries will be noted.

The outcome data will be analysed by multiple regression modelling, with appropriate generalised linear models used to examine the effect of the intervention. The effects reported will be adjusted differences in proportions with confidence intervals, with the adjustment being for site. All subgroup analyses will be specified a priori in the Statistical Analysis Plan (which will be approved by the Data safety and monitoring board (DSMB) before the end of stage 1) and carried out using formal tests for interaction included in the statistical models and assessed for statistical significance using likelihood ratio tests.

The primary analysis will be per-protocol (PP); where patients will be analysed based on the treatment they actually received rather than the one they were allocated to and given the non-inferiority trial design, an intention to treat (ITT) analysis will also to be conducted.

### Interim analyses {21b}

Following completion of stage 1 recruitment, the primary and safety analyses will be provided to the DSMB. The DSMB would then make a recommendation to the SAC as described above. A further interim analysis was planned after 90 patients per arm were recruited into stage 2 of the trial. Stopping was to be considered if a difference between randomised arms of at least 3 standard deviations in the interim analysis of a major endpoint achieved and the results had the potential to impact clinical practice. The final decision would be taken by the Trial Steering Committee based upon a recommendation of the DSMB.

### Methods for additional analyses (e.g. subgroup analyses) {20b}

Subgroup analyses will be performed for the following variables: HIV status, trial site, cavitation on chest x-ray, resistance pattern, previous TB treatment, smear positivity, smoking status, age, sex and SARS-CoV-2 status. Interaction tests between treatment group and the subgroups listed above will be carried out on the additive (i.e. risk difference) scale for the efficacy and safety primary outcomes only. Results for treatment efficacy and safety will be reported, stratified by the factors. Possible reasons for the interaction, such as clinical differences between sites, will be explored. All subgroup analyses will be performed on the ITT, mITT and PP populations.

Additionally, post hoc analyses not originally described in the protocol will be mentioned in the statistical analysis plan.

### Methods in analysis to handle protocol non-adherence and any statistical methods to handle missing data {20c}

For the primary composite outcome, it is assumed that no negative outcome was reached unless one was observed. For culture conversion, it is assumed no culture conversion had occurred if culture conversion was not observed.

A complete case analysis is planned with no imputation.

### Plans to give access to the full protocol, participant-level data and statistical code {31c}

The full protocol and statistical analysis plan will be made available as appendices during the publication of the trial results.

## Oversight and monitoring

### Composition of the coordinating centre and trial steering committee {5d}

The trial is governed by a Steering Committee (SC), an independent Scientific Advisory Committee (SAC), an independent DSMB and the Project Management Team (PMT). The SC’s main responsibility is to provide strategic, political and operational oversight to the trial to ensure the objectives are effectively met within the time frame and resources allotted. The SC approves the protocol and is the decision body for any trial stoppage decisions. The SAC is a committee external and independent from all project collaborators that provides scientific advice to Médecins sans Frontières (MSF) regarding new MDR-TB regimen projects including TB-PRACTECAL. It advises the PMT on the relevance and scientific validity of the trial designs and their implementation. The SAC makes the recommendation on arms to take forward from stage 1 to stage 2. The PMT’s responsibility is translating the project strategic direction and objectives set by the steering committee into a clinical trial that will achieve the intended outcomes. This entails making operational (technical, financial, and administrative) choices and running the day-to-day aspects of the trial.

### Composition of the data monitoring committee, its role and reporting structure {21a}

The DSMB is independent of the sponsor and all project collaborators. It is governed by the DSMB charter which describes its purpose and terms of reference. It consists of a statistician (Chair), a drug development expert, an HIV expert, a TB clinical trials expert and an MDR-TB clinical expert. The overall responsibility of the DSMB is to protect the ethical and safety interests of subjects recruited into the PRACTECAL trial. The committee reviews the accumulating unblinded safety data after every 40 patients recruited to the study or every three months whichever occurs first and meet at least every 6 months. Depending on this evaluation, the DSMB will make recommendations to the SC concerning the continuation, modification, or termination of the study.

### Adverse event reporting and harms {22}

Adverse Events recording applies to both investigational and control arms in the trial. AE recording began upon initiation of study treatment and continued until the patient’s last study visit. All AEs are recorded in the AE section of the eCRF. AEs can be spontaneously reported or elicited during open-ended questioning, examination, or evaluation of a trial participant. The investigator must also promptly review all results of assessments performed as part of the trial, such as laboratory assessment results, ECGs, vital sign monitoring, physical examinations, etc. and assess them for clinically relevant changes compared to baseline. Each AE is evaluated to determine the severity grade: Grade 1–4 as per the latest version of the MSF Severity grading scale [21], its duration (start and end dates or if continuing at the end-of-study visit), its relationship to the study treatment, action taken with respect to study treatment (treatment maintained, dose reduced, permanently discontinued, temporarily discontinued, not applicable), whether medication or therapy was taken/given in relation to the AE and whether it is a serious adverse event (SAE).

### In the study, ICH-GCP definitions for SAE are applied [22]

An adverse event of special interest is one of scientific and medical concern specific to the investigational drug(s), for which on-going monitoring and rapid communication by the investigator to the sponsor is appropriate. Such events require further investigation in order to characterise and understand them. Based on signals observed from previous studies, several AEs of special interest were identified for this trial:

- All grade 4 AEs which are not SAEs
- Grade 3 QT interval prolongation
- Other grade 3 dysrhythmias
- Grade 3 liver enzyme abnormalities (transaminases and bilirubin)
- Any grade of pancreatitis
- Any grade of optic nerve disorder
- Grade 3 peripheral neuropathy
- Any grade of seizures and fainting
- Any grade cataract formation

Every SAE and AE of special interest (AESI) is reported by the investigator to the sponsor’s pharmacovigilance (PV) unit within 24 h of learning of its occurrence. Recurrent episodes, complications, or progression of the initial SAE/AESI are reported as follow-up to the original episode within 24 h of the investigator receiving the follow-up information. Additionally, pregnancy, overdose and malignancy not otherwise serious, require expedited reporting using a similar process.

All adverse drug reactions that are both serious and unexpected are subject to expedited reporting to the National Regulatory Authorities (NRA) and ethics review boards (ERBs). The sponsor is responsible for reporting these events to NRA whilst the site principal investigator is responsible for reporting the events to the local ERB. In the context of this study, reporting to NRAs may be delegated to the sites with close support from the sponsor as detailed in the corresponding SOP.

Fatal or life-threatening suspected unexpected serious adverse drug reactions should be reported as soon as possible and no later than 7 calendar days after first knowledge by the sponsor of the case. Unexpected Serious ADRs that are not fatal or life-threatening must be notified as soon as possible and no later than 15 days after first knowledge by the sponsor of the case. Unless specifically requested by NRAs/ERBs, all SAEs, that are not considered as unexpected ADR are summarised in annual safety reports and submitted to NRA and ERB in due time.

### Frequency and plans for auditing trial conduct {23}

Prior to study start, a Monitoring Plan and Monitoring SOP was developed, agreed upon between the external monitor and the PMT. The site principal investigator will allow the monitors to visit the site and facilities where the study will take place in order to verify compliance with the protocol requirements, ICH-GCP (International Council on Harmonisation – Good Clinical Practice) and WHO-GCLP (World Health Organization – Good Clinical Laboratory Practice). Training sessions on GCP, GCLP and on protocol implementation were organised for the investigators and all study staff prior to recruitment start and as staff join the project. Instruction manuals and SOP will be distributed to all the study centres.

Study monitoring is carried out at regular intervals, depending on the recruitment rate, to verify data quality and study integrity. At the end of each monitoring visit, and based on monitoring visit reports, the PMT will be responsible for controlling recruitment rates, ineligibility, non-compliance, protocol deviations and dropouts overall and in each study centre, completeness and timeliness of data and compliance with GCP, GCLP and applicable regulations

A final monitoring visit will be conducted at the end of the trial, after the last patient, last visit (LPLV), and once the database is locked.

In addition to the planned monitoring activities, the trial may be evaluated by external auditors appointed by the sponsor and by government inspectors who must be allowed access to CRFs, source documents, study files, and study facilities. This will be independent from investigators and sponsors.

### Plans for communicating important protocol amendments to relevant parties (e.g. trial participants, ethical committees) {25}

If the protocol must be altered after it has been signed, the modification or amendment must be discussed and approved by the Principal Investigators and the sponsor. The protocol amendment must be drafted and signed by both parties. All amendments are submitted to the relevant Ethics Committees and NRAs. Administrative amendments can be implemented immediately but amendments that affect other aspects can only be implemented after a favourable opinion of the Ethics Committee and NRA has been obtained and local regulatory requirements have been complied with. An amendment needed to eliminate immediate hazards to the participants in the study is exempted from this rule.

### Dissemination plans {31a}

The results of the trial will be submitted for publication in an open-access peer-reviewed scientific journal and posted in a publicly accessible database of clinical study results within 12 months. Preliminary results will also be shared in global conferences. Communities involved in the study will be informed of the outcomes and other national or global stakeholders will receive relevant information.

## Discussion

TB-PRACTECAL is a multi-arm, multi-stage clinical trial aimed at identifying safe and efficacious regimens to treat rifampicin-resistant tuberculosis. The adaptive trial design was chosen to assess a range of candidate regimens and ensure seamless progression of the most promising regimen/s into phase III. The accelerated model was, if positive, designed to bring a shorter and more efficacious treatment to high-burden communities as soon as practicable. Current MDR/RR-TB treatment remains 9–20 months and carries a significant risk of adverse events. High-quality clinical research for MDR/RR-TB needs extensive resourcing and several years to be able to provide data given the ongoing need for extended follow up.

The trial takes an ambitious and pragmatic approach to regimen advancement compared with earlier explanatory trials into newer tuberculosis drugs such as bedaquiline and delamanid [8, 9, 23]. In doing so, a conservative safety approach is being taken with intensive oversight by the sponsor, regular monitoring from the independent DSMB and continuous pharmacovigilance.

The design features are notable for the continuously updated SOC which has changed radically in all centres since trial inception. This choice complicates the analysis however has aided in ongoing recruitment by ensuring those randomised to SOC will receive the best available treatment at any point in the trial. Patients enrolled in the stage 1 of the trial also continued their treatment arm through to week 108 and their findings will contribute to the stage 2 sample size.

Sites were selected based on a range of factors including differing geography, resource limitations, rates of second-line drug resistance and rates of HIV representing the diversity of contexts and sub-groups most affected by the RR-TB epidemic. Research experience is varied and so a supportive, risk-based monitoring approach was taken and tailored to site needs.

The study aims to add to the research base guiding the use of shorter MDR/RR-TB regimens composed by new and re-purposed drugs. During the study, encouraging results from uncontrolled NIX-TB clinical trial were published [24] and TB-PRACTECAL may complement these findings. Additionally, it may assist in answering whether an additional drug provides added benefit to BPaL regimens and will provide data on an alternative approach to linezolid dosing.

Limitations include limited generalizability to certain populations such as children under 15 and pregnant women who were excluded from entry into the trial. This was an open-label study and blinding was limited to laboratory staff. Outcome assessment was at the investigators discretion but had to be verifiable in the database and in line with the protocol. Any ambiguous outcomes were referred to an independent outcome adjudication committee for final classification. The safety approach meant that patients were discontinued from the trial under conservative rules which were based on the safety profile of the investigational regimens at the trial outset. This may not resemble routine care and limit the strength of the conclusions which can be drawn. However, all arms were handled under the same rules. The effectiveness of any candidate regimen should be further evaluated under programmatic conditions.

## Trial status

The trial is currently operating under protocol version 7.0 or 7.1 (depending on site). The first patient was recruited on 16 January 2017.

Recruitment into stage 1 was completed in mid-2019. The transition procedures were followed per the protocol with all arms meeting the pre-specified eligibility criteria for stage 2. Following the recommendation from the Scientific Advisory Committee to proceed with investigational arms 1 and 2, the steering committee proposed to proceed to stage 2 with arm 1 only. The Sponsor accepted and implemented this decision. Transition from stage 1 to stage 2 was delayed with the COVID-19 pandemic and completed in late 2020. Randomisation into all 4 arms continued until transition was complete at each site.

The COVID-19 pandemic also impacted the trial sites to a varying extent. The Sponsor and sites collaborated to develop a mitigation plan. This allowed increased flexibility given limited patient movements but aimed to minimise impacts on data quality and patient safety. Ensuring continuity of care and treatment, managing infection control risks for staff and patients, and access to care for severe illness or adverse events were key priorities. An earlier switch to the less intensive investigation schedule for stage 2 (pre-dose ECG only, audiometry and slit lamp examinations as clinically indicated only), phone visits, accelerated implementation of VOT at every site and remote monitoring visits were some of the solutions put in place. Slow recruitment was another challenge caused by pandemic: some diagnostic facilities were closed, restriction in movements decreased number of screenings and TB diagnosis and other TB facilities were sometimes repurposed as COVID-19 wards.

In February 2021, the DSMB recommended that the steering committee terminate recruitment based on an observed difference in efficacy between study arms. This advice followed the DSMB charter procedures which recommended that stopping be considered if there was a difference between randomised arms of at least three standard deviations in the interim analysis of a major endpoint. The endpoint also needed to be one that would likely impact clinical practice.

The steering committee followed DSMB recommendations and recruitment ended on the 18th of March 2021. All enrolled patients will continue to be followed up to at least week 72, post-randomisation.

TB-PRACTECAL plans to report data up to date of termination. A revised statistical analysis plan will be adapted for this analysis. The findings will be shared through conference presentations and via submission to a peer-reviewed journal. The trial will continue to follow and monitor the remaining patients through to last patient visit as planned and a final report will also be shared widely.

## Supplementary information

**Additional file 1.**

## Data Availability

The trial data will be made available after the primary publication or twelve months after trial completion (whichever is earlier) upon reasonable request and in agreement with the MSF Data sharing policy.

## Abbreviations

ADR: Adverse drug reaction
AE: Adverse events
AESI: Adverse event of special interest
ALT: Alanine aminotransferase
AST: Aspartate aminotransferase
B: Bedaquiline
CD4: Cluster of differentiation 4 cells
CFR: Code of Federal Regulations
Cfz: Clofazimine
COVID-19: Coronavirus disease caused by SARS-CoV-2 virus
Cs: Cycloserine
DOT: Directly observed therapy
DS: Drug sensitive
DSMB: Data and Safety Monitoring Committee Board
E: Ethambutol
ECG: Electrocardiogram
eCRF: Electronic case report form
EDC: Electronic data capture
ERB: Institutional Ethics Review Board
Eto: Ethionamide
FDA: Food and drug administration
GLM: Generalised linear models
H: Isoniazid
HIV: Human immunodeficiency virus
ICF: Informed consent form
ICH-GCP: International Committee on Harmonisation of Good Clinical Practice
IMP: Investigational medicinal product
INN: International non-proprietary name
ITT: Intention to treat
IQR: Interquartile range
Lfx: Levofloxacin
LIS: Lab information system
LSHTM: London School of Hygiene and Tropical Medicine
Lzd: Linezolid
MDR: Multidrug-resistant
MedDRA: Medical Dictionary for Regulatory Activities
Mfx: Moxifloxacin
MGIT: Mycobacteria growth indicator tube
mITT: Modified intention to treat
MSF: Médecins Sans Frontières
MTB: *Mycobacterium tuberculosis*
NRA: National Regulatory Authority
Ofx: Ofloxacin
Pa: Pretomanid (PA-824)
PAS: Para-amino-salicylate sodium
PMT: Project Management Team
PP: Per-protocol
Pto: Prothionamide
QT: Interval between Q and T waves on an ECG
QTc: QT interval corrected
QTcF: QT interval corrected using Fridericia’s formula
R: Rifampicin
RR-TB: Rifampicin-resistant TB
SAC: Scientific Advisory Committee
SAE: Serious adverse events
SARS-CoV-2: Severe acute respiratory syndrome coronavirus 2
SC: Steering Committee
SD: Standard deviation
SOC: Standard of care
SOP: Standard operating procedure
SUSAR: Severe unexpected serious adverse drug reaction
TB: Tuberculosis
TdP: Torsades de Pointes
Trd: Terizidone
VOT: Video observed therapy
WHO: World Health Organization
XDR: Extensively drug-resistant
Z: Pyrazinamide
