# Supplementary material for: TB-PRACTECAL: study protocol for a randomised, controlled, open-label, phase II–III trial to evaluate the safety and efficacy of regimens containing bedaquiline and pretomanid for the treatment of adult patients with pulmonary multidrug-resistant tuberculosis
Source: Trials. 2022 Jun 13;23:484. doi: 10.1186/s13063-022-06331-8 (PMC9190445; doi:10.1186/s13063-022-06331-8)
Supplement: Supplementary file 1 — Additional file 1. [file 13063_2022_6331_MOESM1_ESM.docx]

|  | **screening** | **Inclusion^o^** | **TREATMENT & FOLLOW-UP** | | | | | | | | | | | | | | | | | | | | | | | | | | | | **early discontinuation^n^** |
| --- | --- | --- | --- | --- | --- | --- | --- | --- | --- | --- | --- | --- | --- | --- | --- | --- | --- | --- | --- | --- | --- | --- | --- | --- | --- | --- | --- | --- | --- | --- | --- |
| **Frequency of visits** |  |  | **every 2 days** | | | | | | | **weekly** | | | | | | **every 4 weeks** | | | | **every 8 weeks** | | | | | | | | | | |  |
| **Visit Number** | **1** | **2** | **3** | **3b** | **3c** | **4** | **4b** | **4c** | **5** | **5b** | **6** | **6b** | **6c** | **6d** | **7** | **8** | **9** | **10** | **11** | **12** | **13** | **14** | **15** | **16** | **17** | **18** | **19** | **20** | **21** | **22** |  |
| **Timing of trial visit** | **<W -4** | **D 0** | **D1** | **D 3** | **D5** | **D 7** | **D 9** | **D 11** | **D 14** | **W 3** | **W 4** | **W5** | **W6** | **W7** | **W 8** | **W 12** | **W 16** | **W 20** | **W 24** | **W 32** | **W 40** | **W 48** | **W 56** | **W 64** | **W72** | **W80** | **W88** | **W96** | **W104** | **W108** |  |
| **Demographics** | x |  |  |  |  |  |  |  |  |  |  |  |  |  |  |  |  |  |  |  |  |  |  |  |  |  |  |  |  |  |  |
| **Past medical / treatment history** | x |  |  |  |  |  |  |  |  |  |  |  |  |  |  |  |  |  |  |  |  |  |  |  |  |  |  |  |  |  |  |
| **Inclusion and exclusion criteria** | x | x |  |  |  |  |  |  |  |  |  |  |  |  |  |  |  |  |  |  |  |  |  |  |  |  |  |  |  |  |  |
| **Informed Consent** | x | x |  |  |  |  |  |  |  |  |  |  |  |  |  |  |  |  |  |  |  |  |  |  |  |  |  |  |  |  |  |
| **Randomisation** |  | x |  |  |  |  |  |  |  |  |  |  |  |  |  |  |  |  |  |  |  |  |  |  |  |  |  |  |  |  |  |
| **Current medical history and Physical examination** | x | x | x | x | x | x | x | x | x | x | x | x | x | x | x | x | x | x | x | x | x | x | x | x | x | x | x | x | x | x | x |
| **Pregnancy test** | x | x^(a)^ |  |  |  |  |  |  |  |  |  |  |  |  |  | x | x | x | x |  |  |  |  |  |  |  |  |  |  |  | x |
| **Hepatitis B and C** | x |  |  |  |  |  |  |  |  |  |  |  |  |  |  |  |  |  |  |  |  |  |  |  |  |  |  |  |  |  |  |
| **HIV test^(b)^** | x |  |  |  |  |  |  |  |  |  |  |  |  |  |  |  |  |  | x |  |  |  |  |  |  |  |  |  |  |  |  |
| **CD4 count and Viral load ^(c)^** | x |  |  |  |  |  |  |  |  |  |  |  |  |  |  |  |  |  | x |  |  |  |  |  | x |  |  |  |  | x | x |
| **Laboratory tests ^(d)^** | x | x^(a)^ |  |  |  | x |  |  | x | x | x | x | x | x | x | x | x | x | x | x |  |  |  |  | x |  |  |  |  | x | x |
| **PK blood sample** |  |  | x^(e)^ |  |  |  |  |  |  |  |  |  |  |  | x^(f)^ | x^(g)^ | x^(g)^ | x^(g)^ | x^(g)^ | x^(g)^ |  |  |  |  | x^(h)^ |  |  |  |  |  |  |
| **PK dry blood sample ^(i)^** |  |  | x |  |  |  |  |  |  |  |  |  |  |  | x | x | x | x | x | x |  |  |  |  | x |  |  |  |  |  |  |
| **PG blood sample** |  |  | x^(j)^ |  |  |  |  |  |  |  |  |  |  |  |  |  |  |  |  |  |  |  |  |  |  |  |  |  |  |  |  |
| **PK hair sample** |  |  |  |  |  |  |  |  |  |  |  |  |  |  | x |  | x |  | x | x |  |  |  |  | x |  |  |  |  |  |  |
| **TSH** | x | x^(a)^ |  |  |  |  |  |  |  |  |  |  |  |  |  |  |  |  | x |  |  |  |  |  | x |  |  |  |  | x | x |
| **12-lead triplicate ECG – pre-dose^(k)^** | x | x |  |  |  | x |  |  | x | x | x | x | x | x | x | x | x | x | x | x | x | x |  |  | x |  |  |  |  | x | x |
| **12-lead triplicate ECG – post dose** |  |  |  |  |  | x |  |  | x | x | x | x | x | x | x |  |  |  |  |  |  |  |  |  |  |  |  |  |  |  |  |
| **Sputum smear^(l)^** | x^(p)^ | x |  |  |  | x |  |  |  |  | x |  |  |  | x | x | x | x | x | x | x | x | x | x | x | x | x | x | x | x | x |
| **Sputum culture^(l)^** |  | x |  |  |  | x |  |  |  |  | x |  |  |  | x | x | x | x | x | x | x | x | x | x | x | x | x | x | x | x | x |
| **DST^(l)^** |  | x |  |  |  | x |  |  |  |  | x |  |  |  |  |  | x | x | x | x | x | x | x | x | x | x | x | x | x | x | x |
| **MIC (Lzd, B, Pa) ^(l)^** |  | x |  |  |  | x |  |  |  |  |  |  |  |  |  |  | x | x | x | x | x | x | x | x | x | x | x | x | x | x | x |
| **WRDT** | x |  |  |  |  |  |  |  |  |  |  |  |  |  |  |  |  |  |  |  |  |  |  |  |  |  |  |  |  |  |  |
| **Chest X-Ray** | x |  |  |  |  |  |  |  |  |  |  |  |  |  |  |  |  |  | x |  |  |  |  |  |  |  |  |  |  |  | x |
| **Slit lamp exam** | x |  |  |  |  |  |  |  |  |  |  |  |  |  |  |  |  |  |  |  | x |  |  |  |  |  |  |  |  |  | x |
| **Ophthalmology assessment** | x |  |  |  |  | x |  |  | x |  | x |  | x |  | x | x | x | x | x |  | x |  |  |  |  |  |  |  |  |  | x |
| **Audiometry** | x |  |  |  |  |  |  |  |  |  |  |  |  |  | x |  |  |  | x |  |  |  |  |  | x |  |  |  |  |  | x |
| **Adverse Events** |  |  | x | x | x | x | x | x | x | x | x | x | x | x | x | x | x | x | x | x | x | x | x | x | x | x | x | x | x | x | x |
| **Concomitant treatments^(m)^** |  |  | x | x | x | x | x | x | x | x | x | x | x | x | x | x | x | x | x | x | x | x | x | x | x | x | x | x | x | x | x |
| **Treatment compliance^(m)^** |  |  |  |  |  | x |  |  | x | x | x | x | x | x | x | x | x | x | x | x | x | x | x | x | x | x | x | x | x | x | x |
| **Covid-19 serology** | x |  |  |  |  |  |  |  |  |  |  |  |  |  |  |  |  |  | x |  |  |  |  |  | x |  |  |  |  |  |  |

Table 3 stage 1 investigational schedule

^a)^ Not necessary if there is delay of less than 1 week ( 3 days for pregnancy test and medical history) between the day the screening test was done and the day randomisation is done.

^b)^ Done only if the patient is not known to be HIV positive and no test result is available from the past 1 calendar months

^c)^ Only for HIV positive patients

^d)^ Includes full blood count and differential, LFTs (ALT, AST, Total bilirubin, ALP), chemistry (sodium, potassium, creatinine, BUN), lipase, total protein, glucose only at screening and if symptoms.

^e)^ Collected in Lithium heparin tubes at 0, 2 and 23 hours post dose

^f)^ Collected in Lithium heparin tubes at 0, 6.5 and 23 hours post dose

^g)^ Collected in Lithium heparin tube within 30 min pre-dose

^h)^ Collected in Lithium heparin tube if feasible

^i)^ Collected from venous blood and capillary onto a Mitra device once a day

^j)^ Collected in an EDTA tube at earliest opportunity

^k)^ Predose ECG to be done before any other test or exam is conducted on the patient

^l)^ Two sputum samples collected early morning and on-the-spot coached on the day of collection. Conventional DST to at least R, H, Ofx, Km, Cm will be done on culture positive samples at baseline and from 16 weeks. Isolates will then be stored for MIC and /or strain typing.

^m)^ SOC only for week 32 to week 108

^n)^ Repeat test only if not done already within the past 28 days. Tests can also be done within 7 days after early discontinuation

^o)^ Randomisation may take place a day before the inclusion visit

^p)^ This is a historical sample and recorded, when available, and performed according to local procedures
